# Medical Association of Georgia

**Published:** 1875-05

**Authors:** 


### MEDICAL ASSOCIATION OF GEORGIA.

### FIRST DAY.

SAVANNAH, April 21, 1875.

Association convened at the Armory Hall, at 10 A.M., about sixty members present, and was opened in a brief address of welcome by Dr. R. D. Arnold, the venerable nestor of the Savannah profession, who displayed much of his former vigor of mind and body, notwithstanding advancing age. By designation of the President, Dr. H. F. Campbell of Augusta responded very briefly in behalf of the assembled members.

Dr. DeS. Ford, the President elect, delivered his inaugural address. He considered the subject of the antagonism between science and theology—evidencing a goodly familiarity with his subject—the general sentiment of the address being that science and theology each has its field of useful labor, and there need not be, as there should not be, any conflict between the two.

The President appointed Drs. H. F. Campbell, Juriah Harris and T. J. Word to fill vacancies on Board of Censors for the session. Dr. Harris of Savannah introduced a resolution inviting to seats on the floor visiting members of the profession, medical officers of the army and navy, and members of the city press. Adopted.

Dr. Juriah Harris, chairman of the Committee of Arrangements, announced the programme for the entertainment of the members as follows:

Wednesday evening, receptions at the residences of Drs. Thomas, Harris and Charlton, from 8 to 11 o'clock. An excursion to the sea on Thursday afternoon. Friday afternoon, an excursion by rail to the Isle of Hope.

Dr. W. F. Westmoreland introduced a resolution, which was adopted, requiring all voluntary communications and reports of congressional committees to be read by title, to be called up at the pleasure of the Association. Under the call for voluntary communications, the following papers were introduced: A report of twelve cases of opium poisoning, by E. J. Roach, M.D., Atlanta; Urethral dilatation on the multiple wedge principle, by John S. Coleman, M.D., Augusta; Iridotomy, its applicability to certain defects of the eye, with a report of four cases, by A. W. Calhoun, M.D., Atlanta; New uterine stem, secured by means of a silver stitch through the cervix, by V. H. Taliaferro, M.D., Atlanta; The influence of yellow fever upon pregnant and parturient women, with a report of cases, by A. J. Semmes, M.D., Savannah; The treatment of tetanus by chloral hydrat, by Theo. Scott, M.D.; The possibilities of the hypodermic syringe, by DeSaussure Ford, M.D., Augusta; Nutritious enemata as a means of sustaining life, by A. Sibley Campbell, M.D., Augusta; Blood-letting, by C. B. Nottingham, M.D., Macon; Carbolic acid as a local anæsthetic in surgical operations, by Eugene Foster, M.D., Augusta; Anaplexia of pneumonitis, by J. E. Susdorff, M.D., Macon; Report of four cases of hydatid degeneration of the ovum, by R. C. Eve, M.D., Augusta; Successful amputation of thigh for compound, comminuted fracture of middle third, several weeks after injury, by Robert Norton, M.D., Effingham county.

The report of the Board of Censors, recommending the acceptance of the resignation of J. A. Urquhart, M.D., was adopted.

Dr. Word introduced a resolution, which was adopted, expressing the esteem in which Dr. Urquhart was held, and regret at his withdrawal from the Association.

On motion of Dr. W. F. Westmoreland, the voluntary communications were called up and read in the order in which they were presented.

Dr. Roach being absent, the reading of his paper was deferred.

The paper by Dr. Taliaferro was read, and specimens of the uterine stem exhibited. He called attention to two kinds of uterine stem: one composed of a section of a steel watch spring, enveloped in cloth and coated with gutta percha, and used for its mechanical effect simply; the other, the galvanic stem, consists of a strip of zinc and a strip of copper riveted together. These stems are retained in the uterus by silver wires passed through the lower end and through one side of the cervix uteri. The wire is secured by shot clamped on each end, and is entirely unirritating to the tissues.

Dr. Theo. Starbuck's paper, on the treatment of tetanus by chloral hydrat, was read. He reported several cases treated by hydrate of chloral, either alone or in conjunction with bromide potassium, or opium. In one a small quantity of chloroform by inhalation preceded the administration of the chloral. All the cases recovered. Considers it important to give the chloral regardless of the amount, but regulates the quantity by the effect on the spasm. In one of his cases he gave five and a half ounces in eleven days. The spasms were controlled, but no drowsiness was produced.

Dr. A. W. Calhoun read his paper on Iridotomy and its applicability to certain affections of the eye. This operation is now used largely as a substitute for the old operation of iridectomy. The former term signifies cutting into the iris, the latter cutting a portion out of the iris. It is indicated when a small artificial pupil is desired. The opening after iridectomy is often so large that the quantity of light admitted confuses vision. Iridotomy is simply a slit into the iris, made by a pair of scissors, and the contraction of the circular fibres is sufficient to produce a wedge-shaped opening, having its base towards the pupil. The closed blades of the scissors are introduced through an incision made by a spear-shaped knife at the corneo-sclerotic junction. Great care is required to avoid wounding the lens or its capsule, on which one blade of the scissors must rest in the operation.

### FIRST DAY--AFTERNOON.

Dr. C. B. Nottingham, of Macon, read an essay upon Blood-letting, in which he reviewed the ground upon which rests the present disuse of the lancet, and taking position in favor of a discrete and enlightened use of this potent, and often most effective, agent for the stay of the violence of disease. He combatted the idea of the change of type of disease, as urged by many, as a reason for the entire abandonment of the lancet. This agent was, in former times, doubtless greatly abused, but the present entire disuse of venesection is not consistent with a truly enlightened and effective therapia. He reviewed the conditions in which the lancet affords most effective aid in pneumonia and other phlegmasiæ; also in the maladies of pregnant, parturient and puerperal women.

Dr. Eugene Foster read an essay upon carbolic acid as a local anæsthetic in surgical operations. He admits that is a local anæsthetic, but has found it to be escharotic also, as the skin dies and is thrown off; and it is curious to notice also that it is followed by no suppuration during the process of cicatrization. It produces great pain in whitlow. Regards it, as a local anæsthetic, to be utterly worthless.

Dr. Eve read a paper for John L. Coleman, M.D., of Augusta, on the treatment of stricture on the multiple wedge principle. The advantage, he contends, over the ordinary conical bougie is that the wedge is introduced in sections, and consequently the resistance is limited to a single point, and does not include the whole circumference of the constriction. Uses quinine during the treatment of stricture as a prophylactic against urethral fever.

In the course of discussion on this paper, Dr. H. F. Campbell of Augusta said he had always regarded cutting a stricture and perineal section as an absurdity until he became acquainted with Sir Henry Thompson's views. He always, until now, held that when the smallest instrument could be introduced he was safe, and a cure was insured by gradual dilatation. But Sir Henry Thompson urges, in view of the liability to return even after months of tedious treatment, that certain strictures had better be cut.

Dr. Coleman offers his plan for those cases in which, by ordinary methods, excepting perineal section, relief would not be sufficiently speedy to rescue the patient.

---

SECOND DAY.

Meeting called to order by the President.

On motion of Dr. O'Daniel, a committee of three was appointed to examine the books and vouchers of the Treasurer. T. F. Walker, Geo. A. Wilcox and Jas. B. Baird, committee.

Dr. J. P. Logan called attention to the absence of any law in this State for the punishment of fœticide, and read a memorial and draft of a bill presented by the Atlanta Academy of Medicine to the last Legislature. The bill failed. He therefore moved that the Association indorse this effort, and appoint a committee to bring the question before the Legislature at its next session. Adopted. J. P. Logan, William Duncan, Eugene Foster, A. H. Kennan and B. S. Doster, committee.

The report of Drs. A. W. Griggs and W. S. Kendrick, delegates to the Medical Association of Alabama, was received.

Dr. W. F. Westmoreland referred to the failure of the passage of the bill by the last Congress, relating to the rank and pay of the medical officers of the United States Army. He offered a preamble and resolutions, which were adopted, deprecating the failure of Congress to act in this matter, and providing for the appointment of a committee to urge upon Congress the passage of the bill; that a copy of the resolutions be sent to the American Medical Association, and that it and local societies be requested to co-operate in this endeavor to secure justice towards a meritorious department of the public service.

Dr. A. J. Semmes, of Savannah, read a paper on the influence of yellow fever upon pregnant and parturient women; indicating the disposition to abortion and premature labors, and the general opinion of physicians of the extreme fatality attending abortions and labors during an attack of yellow fever. The well known and universal law, that hæmorrhage from the uterus and vagina are always present in women of the menstruating age, commencing about the end of the second or third day, during an attack of yellow fever; and the danger which attends the exposure of pregnant women to yellow fever, and the necessity of removal from localities where the epidemic prevails.

The nominating committee recommended the following officers for the ensuing year, and the report was unanimously adopted, viz: President, J. G. Thomas, M.D., Savannah; First Vice-President, T. J. Word, M.D., Columbus; Second Vice-President, W. A. Greene, M.D., Americus; Censor, T. J. Charlton, M.D., Savannah.

Augusta was unanimously selected as the next place of meeting.

Dr. Ford read a paper by Dr. L. A. Dugas, on mania-a-portu, recommending for its treatment cold douches to the head and entire body, not to be continued for more than three minutes. Does not use narcotics.

Dr. Rob't P. Myers, Savannah, read the history of a case of fistula-in-ano treated by the local use of a solution of sulphate of quinine, gr. x to water ℥j., continued three weeks, with perfect cure.

Also a case of resuscitation from chloroform poisoning, by M. Nelaton's method; a case of amputation of arm, recovering in four weeks; stump dressed with carbolized oil, i.e., carbolic acid, ʒij; boiled linseed oil, ℥viij. Also a case of a pachydermatocele of the left labium majus of unusual size; weight 9½ pounds; reached to the knee. Wound healed, but death occurred in one month, from broken-down constitution.

Dr. Purse read a paper; Dr. Word also.

---

THIRD DAY.

Association convened, the President in the chair.

The committee appointed to audit the Treasurer's accounts reported that they found them correct.

The report of the committee appointed at the preceding meeting, to revise the by-laws, was amended and adopted.

A resolution was introduced by Dr. Eugene Foster, and adopted, providing, in the event the funds of the Association did not warrant the publication of the transactions in the usual form, that the authors of the papers be allowed to publish them in any medical journal.

Dr. Rauschenberg, of Atlanta, chairman of the Committee on Hygiene, read his report, explaining the significance and importance of hygiene, its present status in the United States and in Georgia, and the relation which the medical profession and medical organizations occupy towards it. He elucidated the great influence of sanitary investigations and sanitary laws and regulations upon the physical, intellectual and moral progress of communities and populations, and the great duty of the medical profession, not only to inquire into the nature and treatment, but also into the cause and mode of prevention of disease, so that the mortality of mankind might be decreased, and its integrity, physically, mentally and morally, improved. He congratulated the State on her acdnowledgment of the duty of nct only protecting the lives of her citizens against the assassin and murderer, but also against the manifold causes of disease uncontrollable by individuals, and only amenable to proper sanitary laws and regulations, such as Dr. Thomas' late law, creating a State Board of

Health, initiates. He finally illustrated the nature of the task now before the profession and medical societies in relation to sanitary service, and expressed the wish that the Georgia Medical Association might keep this task permanently before her, and might succeed in establishing all the sanitary reforms, in city, town and country, which are required for the welfare of her inhabitants, and that her citizens, in recognition of the benefits of these measures, might soon become firm advocates and zealous friends of sanitary legislation.

A communication from Dr. A. Means was read, on the chemical composition and antiseptic properties of salicylic acid.

Dr. Rauschenberg, of Atlanta, introduced a resolution of thanks to Dr. J. G. Thomas for his services in superintending the passage of the law creating a State Board of Health. Unanimously adopted.

Also a resolution requesting the State Medical Board to zealously prosecute its labors in examining druggists, and that the press of the State be requested to publish the resolution. Adopted.

A paper by Dr. Schaffer, of Lawrenceville, on Imperforate Hymen, and other obstructions to menstruation, was called up and read.

Dr. Eve, of Augusta, read an essay on the Influence of the Ovaries in Uterine Disease.

Dr. H. F. Campbell, chairman of the Committee on Gynæcology for the Eighth Congressional District, presented his report. He said: Every sexual abnormality may present two momentous desiderata, the health of the patient and the relief of sterility; both ends are compromised by uterine displacements. Chains of nervous connection bring the uterus into close relationship with other parts of the body. Hence, in many instances mental depression accompanies sterility. A displaced womb, free from disease, would be a typical case of uterine displacement. It matters not how pressed upon and distorted, to replace and retain the organ would be the simple and only duty of the physician. But these are not the kind of cases usually encountered in practice. The complications met with present the most serious obstacles to retention in situ; such as increased size, weight, ulceration, polypoid excrescences, and many other conditions, so that replacement is unnecessary, as retention is impossible until these most troublesome complications are removed.

To effect replacement, the knee-and-breast posture offers the most effectual means, and its range of applicability is wider than any other means that can be employed. This position had its existence as a remedy in the early part of the present century, and was used for replacing the prolapsed funis.

Dr. Marion Sims has particularly described it, and in his earlier experience attached great importance to it in operations for vesico-vaginal fistula. He, however, now substitutes the semi-prone position. The semi-prone posture is most convenient for certain operations, but is not effective in many conditions that Sims in describing it did not contemplate. The most valuable application of the knee and breast posture is its applicability for replacement of the uterus occupying any mal-position. It is the only proper position in which to attempt the replacement of the uterus; by it gravity is reversed. But this position alone will not replace the womb. Pneumatic pressure in the vagina is an indisputable element in the process. The labia should be separated to enable the air to enter, and it will be found that a sufficient amount will enter, even through a very narrow opening in the hymen. A great advantage of the method is it places the means of relief in the hands of the patient herself, and if practiced every night, and perseveringly, is very efficient. He alluded to the use made of this method by the late Dr. Milton Anthony, of Augusta as early as the year 1836.

The allusion of Dr. Campbell, called forth from Dr. Battey, of Atlanta, a feeling tribute to the memory of Milton Anthony, the friend and counselor of his parents, whose fostering hand had opened for the first time his eyes to the light of day, and conducted him safely through the manifold dangers of puling infancy. A man who had in his useful life endeared himself to the community he served, and to the learned profession which he adorned.

Dr. Nottingham offered a series of resolutions looking to the future organization, by the United States Congress, of a National Health Council. He deprecated the attachment of a mere health bureau to one of the departments, and desired the creation of a separate department, devoted exclusively to this great and most important work of sanitary science.

Drs. Westmoreland and Campbell spoke in support of the measure.

Dr. Foster, of Augusta, introduced a resolution calling for the appointment of a committee of three to again memorialize the

Legislature of Georgia for the establishment of an Inebriate Asylum in the State, which was supported in brief addresses by Drs. Campbell and Eve. Adopted. Committee, Drs. Foster, Meyers and Nottingham.

Dr. Kolloch moved the appointment of a committee to memorialize the Legislature for the legalization of dissections in Georgia. Adopted.

Dr. Robert P. Myers moved the appointment of a committee to draft a form for certificates of membership, and design for a seal for the Association. Adopted. Committee, Drs. Myers, Battey and Wilcox.

The President appointed the following delegates to the American Medical Association, namely: Drs. T, F. Walker, of Cochrane; W. O'Daniel, of Twiggs county; E. J. Kirkscey, F. A. Stanford, T. J. Word and T. S. Tuggle, of Columbus; Robert Battey, W. F. Westmoreland, W. A. Love, G. G. Crawford and J. T. Johnson, of Atlanta; R. D. Arnold, Juriah Harris, R. P. Myers, J. G. Thomas, R. H. Lewis, of Savannah; G. E. Susdorff, W. R. Burgess, W. F. Holt and C. B. Nottingham, of Macon; H. F. Campbell, L. A. Dugas, R. C. Eve and W. H. Doughty, of Augusta; T. S. Hopkins, of Thomasville; G. W. Holmes, of Rome; B. R. Doster, of Blakely. To which, on motion, the name of the President was added.

On motion of Dr. Battey, of Atlanta, the usual resolutions of thanks were unanimously adopted; and after a brief farewell from the President, the Association adjourned to the meeting in Augusta next year, on the third Wednesday in April.

